# The role of juvenile hormone in regulating reproductive physiology and dominance in *Dinoponera quadriceps* ants

**DOI:** 10.7717/peerj.6512

**Published:** 2019-03-01

**Authors:** Victoria C. Norman, Tobias Pamminger, Fabio Nascimento, William O.H. Hughes

**Affiliations:** 1 School of Biology, University of Leeds, Leeds, United Kingdom; 2 School of Life Sciences, University of Sussex, Brighton, United Kingdom; 3 Departamento de Biologia, Universidade de São Paulo, Ribeirão Preto, Brazil

**Keywords:** Reproductive skew, Social insect, Social hierarchy, Social dominance, Eusociality

## Abstract

Unequal reproductive output among members of the same sex (reproductive skew) is a common phenomenon in a wide range of communally breeding animals. In such species, reproductive dominance is often acquired during antagonistic interactions between group members that establish a reproductive hierarchy in which only a few individuals reproduce. Rank-specific syndromes of behavioural and physiological traits characterize such hierarchies, but how antagonistic behavioural interactions translate into stable rank-specific syndromes remains poorly understood. The pleiotropic nature of hormones makes them prime candidates for generating such syndromes as they physiologically integrate environmental (social) information, and often affect reproduction and behaviour simultaneously. Juvenile hormone (JH) is one of several hormones that occupy such a central regulatory role in insects and has been suggested to regulate reproductive hierarchies in a wide range of social insects including ants. Here we use experimental manipulation to investigate the effect of JH levels on reproductive physiology and social dominance in high-ranked workers of the eusocial ant *Dinoponera quadriceps*, a species that has secondarily reverted to queenless, simple societies. We show that JH regulated reproductive physiology, with ants in which JH levels were experimentally elevated having more regressed ovaries. In contrast, we found no evidence of JH levels affecting dominance in social interactions. This could indicate that JH and ovary development are decoupled from dominance in this species, however only high-ranked workers were investigated. The results therefore confirm that the regulatory role of JH in reproductive physiology in this ant species is in keeping with its highly eusocial ancestors rather than its secondary reversion to simple societies, but more investigation is needed to disentangle the relationships between hormones, behaviour and hierarchies.

## Introduction

In many group-living animals, reproduction is not equally distributed among the breeding members. This phenomenon, known as reproductive skew, occurs in a wide range of communally-breeding species including birds, fishes and insects (Jamieson, 1997; Cuvillier-Hot et al., 2004; Neff, Pitcher & Ramnarine, 2008). Due to its fundamental implications for both ecological and evolutionary processes, this topic has attracted attention over the past decades from both a theoretical (see Kokko & Johnstone, 1999; Johnstone, 2000; Kokko, 2003) and empirical perspective in a variety of study systems (Bourke, Green & Bruford, 1997; Field et al., 1998; Reeve & Keller, 2001; Widdig et al., 2004).

Social insects, in particular ants, have emerged as important model systems to test some of the main predictions of reproductive skew theory due to their wide range of social complexity and life history strategies (Reeve & Keller, 2001). The majority of ant species are highly eusocial, with complex societies in which one or several queens produce all female offspring and her unmated daughter workers perform all other tasks such as brood care, foraging and nest defence (Hölldobler & Wilson, 1990). In such systems, as a result of the haplodiploid sex determination in Hymenoptera, workers are only able to produce male offspring and conflicts between queens and workers, or between workers, arise over male parentage (Ratnieks & Reeve, 1992; Ratnieks, Foster & Wenseleers, 2006). This group conflict and how it is resolved has generated a plethora of ground-breaking work, revolutionizing our understanding of group formation, conflict and maintenance (Ratnieks, Foster & Wenseleers, 2006).

While most modern ants have a specialized queen caste, some genera, such as *Dinoponera*, have secondarily reverted to simple, queenless societies in which reproduction is monopolized instead by mated, reproductively active workers called gamergates (Peeters, 1997). In most gamergate systems, all workers have the potential to become the dominant reproductive, resulting in strong within-group conflict over reproduction (Peeters, 1997). Such conflicts are often resolved via aggressive behavioural interactions that establish a dominance hierarchy in which only a single, or a small group of workers go on to reproduce. The question of how such ritualized physical aggression is physiologically translated into stable reproductive hierarchies with lower ranked workers not only remaining reproductively inactive but also assuming helper roles, remains poorly understood. In some species such as *D. quadriceps*, subordinate workers play a role in stabilizing the dominance hierarchy (Monnin & Peeters, 1999; Monnin et al., 2002).

Hormones are prime candidates for the proximate mechanisms underlying this process, because they not only physiologically integrate social stimuli including stress, but also regulate numerous other essential processes in adult insects such as reproduction, maternal behaviour and aggression (Nijhout, 1998; Sasaki, Yamasaki & Nagao, 2007; Tibbetts & Huang, 2010). There are several hormones that have been implicated in this, including dopamine, ecdysone and vitellogenin, with juvenile hormone (JH) being perhaps the best studied. However, it appears that JH can have contrasting effects in different taxa. In primitively eusocial species, such as paper wasps and bumblebees, JH is gonadotropic, stimulating ovary development in the same way as in solitary insects and resulting in individuals being more socially dominant, while in the highly eusocial honeybee, in contrast, JH has lost its gonadotropic effect and instead is involved in regulating division of labour (Robinson & Vargo, 1997). In highly eusocial *Lasius niger* ants, the gonadotropic effect of JH has not only been lost but reversed, with higher JH levels being associated with reduced egg production (Pamminger et al., 2016; Pamminger, Treanor & Hughes, 2016). In addition JH has been shown to trigger foraging behaviour in some ants, making it a possible candidate to coordinate not only reproductive division of labour, but also division of labour between workers (Robinson & Vargo, 1997; Norman & Hughes, 2016). This might suggest a relatively simple switch in the action of JH with the evolution of complex eusocial societies, but it appears that the evolution dynamics of JH mode of action is more complex than that. In *Solenopsis* and *Pogonomyrmex*, ant genera with complex societies, JH exhibits stimulatory functions during reproduction (Brent & Vargo, 2003; Libbrecht et al., 2013). In *Streblognathus* and *Diacamma*, ants with simple, queenless societies, low JH titres in gamergates correlates with high individual ranks within the hierarchy and JH application will result in a loss of the reproductive status of the alpha (Sommer, Hölldobler & Rembold, 1993; Cuvillier-Hot, Lenoir & Peeters, 2004; Brent et al., 2006). In *Harpegnathos* ants, which also have simple, societies, where gamergates can reproduce following the founding queen’s death, JH levels do not differ between reproductive and non-reproductive individuals, and experimental elevation of JH levels through the application of Juvenile Hormone analogue had no effect on egg production (Penick, Liebig & Brent, 2011).

Here we investigate the effects of JH on reproductive physiology and social dominance in the queenless ponerine ant *Dinoponera quadriceps*, by using topical application of the JH analogue (JHa) methoprene to experimentally manipulate JH levels. This species is of particular interest because it is one of ca. 100 species to have undergone an evolutionary reversion from a highly eusocial ancestor with a queen caste back to its basal state with queenless, simple societies (Peeters, 1997; Monnin & Peeters, 1998). All females in *D. quadriceps* are morphologically identical, with a single dominant gamergate, the alpha, actively suppressing a group of the higher ranked workers from becoming reproductively active with ritualized physical aggression including antennal blocking and boxing (Monnin & Peeters, 1998; Grainger et al., 2014). The presence of an alpha within the colony not only inhibits ovary activation in workers, the first step towards becoming reproductively active, but also results in submissive behaviour by subordinates (Smith et al., 2011; Asher et al., 2013). The physiological phenotypic differences between alphas and subordinates result from subtle differences in transcriptional network organisation, involving both conserved and novel genes (Patalano et al., 2015). If JH functions similarly to other queenless ant species, then we predict JH will suppress ovarian development and cause high ranking workers to decrease in status. If JH functions as a gonadotropin, similar to solitary insects and social wasps, then we predict JH to activate ovarian development and potentially to move up in the hierarchy. We focus for our experimental manipulation on high-ranked, but not alpha, workers because these have both the potential to move up the hierarchy to become reproductives and the potential to lose their position in the social hierarchy and become middle or low-ranked workers. We measure the effect of JH manipulation on ovary development and dominance behaviour. If JH links reproduction and hierarchy-related behaviours it would then provide a proximate physiological explanation for rank-associated trait syndromes.

## Methods

We used 13 colonies of *D. quadriceps,* which were collected from Bahia state, Brazil in November 2014 under permit from Instituto Brasileiro do Meio Ambiente e dos Recursos Naturais (IBAMA; 14BR004553). All colonies were maintained in the lab at 27 °C and 80% relative humidity for at least six months before the experiment. Colonies were fed with *Tenebrio molitor* larvae and apple, and had *ad libitum* access to water. Each individual was uniquely marked on the pronotum with numbered tags.

### Establishing the dominance hierarchy

Firstly, to establish the dominance hierarchy, colonies were monitored daily for two weeks, with the behaviours and locations of each individual being recorded once each day for 14 days. Observations lasted until each individual per colony had been recorded. Individuals showed high levels of consistency in behaviour and location during this period. Given the positive association in this species between an individual being of high rank and it interacting with brood (Monnin & Peeters, 1999; Asher et al., 2013), any individual that was observed at least once interacting with brood over the 14 day initial observation period was selected to undergo pairwise isolated dyadic interactions to narrow down their position in the social hierarchy. This method pairs every combination of ants sampled to observe which individual in each dyad is the dominant and which the subordinate, based on a characteristic dominance behaviour; this has previously been shown to be a reliable and robust way to establish dominance hierarchies in this species (Grainger et al., 2014). For this, individuals were taken from their colonies and placed individually in pots (85 mm × 75 mm × 55 mm) and allowed to acclimatise for 15 min. Pairs of ants were then placed in a new pot, their dominance interaction was observed and the dominant ant was recorded. This is indicated by only one behaviour in this context: dominant ants stand tall with their antennae either side of the subordinate individual which has antennae laid flat back behind their head (Grainger et al., 2014). This reaction normally occurs within the first 60 s of contact between pairs when it is expressed. We then ranked individuals based on the number of times they expressed dominance and assigned ranks to each individual. The higher-ranking individuals that ranked directly below the alpha and clearly above the remainder of the colony were then selected for the study (two or three high-ranking workers per colony, and 32 in total). Of these, 16 high-ranking workers were treated with a JH analogue (at least one per colony) and 16 as controls (at least one per colony; see below).

### Worker size and weight

Before the start of the experiment all selected workers were immobilized on ice for 1 min and their head width (maximal interorbital distance) measured as proxy for body size, as well as their fresh weight using a Precisa 125A balance.

### Behavioural measures and experimental procedure

Behavioural observations were made daily for five days before the first application of treatments to determine how consistent ants were for a number of behavioural variables. In total, observations took roughly 3–4 h per day, until each individual per colony had been recorded. We carried out daily scans for five days prior to treatment in which we recorded for each focal ant whether or not it was showing any aggression (either within the nest to conspecifics or gaping it’s mandibles in defence outside of the nest), whether or not it was showing any brood care behaviours, and two measures of ‘sociability’: the distance to the nearest ant and the number of ants within 5 cm of the focal ant (‘contacts’). During the same time interval we carried out individual-level assays for activity level, ‘boldness’ and defensive aggression, with the expectation that high rankers would show low activity level (Monnin, Ratnieks & Brandão, 2003), low boldness (as they are based inside and away from any ‘risky’ tasks such as nest defence or foraging (Nascimento et al., 2012; Asher et al., 2013)), and high levels of aggression (known to be associated with higher ranks (Monnin & Peeters, 1999; Cant, Llop & Field, 2006). General activity level was determined simply by placing the focal ant in a 90 mm Petri dish lined with filter paper, leaving it to acclimatise for 2 min, and then videoing the ant for 5 min using a Logitech c920 webcam. Speed of movement was quantified from videos using AntTrak path analysis software (Tranter et al., 2014). ‘Boldness’ was determined by placing the focal ant in a 90 mm Petri dish lined with filter paper and half blackened out with tape across the lid and sides, leaving it to acclimatise for 2 min, and then videoing the ant for 5 min to allow the proportion of time spent in the light half of the Petri dish to be calculated (less bold ants spend more time hiding in the darkened area of the Petri dish). Defensive aggression was determined by placing the focal ant in a pot (85 mm × 75 mm × 55 mm), leaving it to acclimatise for 5 min, and then tapping it gently on the head with the tip of a toothpick, as in Pamminger et al. (2014). The reaction of the ant was ranked (0 = ignore, 1 = antennate, 2 = gape mandibles in a threat response, 3 = bite).

Following the initial assessment of individual behaviour, ants were assigned randomly to either the methoprene treatment or acetone solvent control (CoA) (with at least 1 methoprene treated and 1 control treated ant per colony), and all subsequent behavioural observations were conducted with the observer blind to treatment. For the methoprene treatment, a dose of 16.5 µg of methoprene (PESTANAL®; Sigma Aldrich, St. Louis, MO, USA) in 5 µl acetone was applied to the pronotum three times over a period of 1 week; control ants received 5 µl acetone on the same occasions. This dose was determined during a preliminary experiment and is low compared to the amounts used in other social insect studies (Table S1), indicating that the observed effects are not caused by potential toxic effects of JH at high doses. After two days of acclimatisation post-treatment, we repeated the behavioural observations. We carried out the assays daily for 4 days and on the 5th day carried out dyadic interaction assays between the focal ants and all other workers that had been observed performing brood care behaviour over the past three weeks. This enabled us to determine if the methoprene treatment had not only affected behaviour but also the position of the focal high rank ants in the hierarchy. Following the dyadic interactions, ants were freeze-killed in liquid nitrogen and stored at −80 °C until ovary dissection.

### Ovary dissection and fertility estimates

Ant ovaries were dissected under a Leica S8AP0 stereo microscope and the ovaries were transferred into Ringer solution. The ovaries were photographed using a Leica DFC 295 Camera and the Leica application suite software v. 4.1.0. Three ovarioles were randomly selected for further analysis to keep consistency between individuals. Using a Pyser-SGI® S78 stage micrometer 1.0/0.01 mm and the software ImageJ 1.47v, we measured the minimum, maximum and average width of the third of the ovarioles closest to the oviduct (containing the most developed eggs if present) and the number of vitellogenic eggs, which are the white (yolk), non-transparent and non-deformed portion of the eggs found in the ovarioles.

### Statistical analysis

For the fertility analysis we carried out individual Wilcoxon-signed rank tests for each of the measures of fertility (minimum, maximum and average ovariole width and number of vitellogenic eggs) as response variables against treatment (either methoprene or acetone control (CoA)).

For the behavioural statistical analysis, we used the programme PRIMER 6, version 6.1.13, + add-in, version 1.0.3 (PRIMER-E Ltd.) to perform permutational multivariate analysis of variance (PERMANOVA). PERMANOVA is a non-parametric MANOVA, which has the advantage that it is free from assumptions on data distributions (Anderson, Gorley & Clarke, 2008). All tests were carried out using 9,999 permutations on a resemblance matrix using Euclidean distance as a distance estimate. In all cases we used treatment as a fixed factor and colony as a random predictor variable to account for the structured nature of the data. Interaction between the factors was included, but removed from the final minimum adequate model when nonsignificant. All response variables were z-transformed prior to treatment in order to account for difference in units and variation between variables, which facilitates the interpretation of results in particular interactions between variables (Gotelli & Ellison, 2004).

To test for potential differences in weight and size between workers belonging to different colonies and treatments, both were used as response variables in a PERMANOVA. We also used PERMANOVA to investigate the effects of treatment and colony on fertility and behaviour. We used the change in behaviour following JH treatment as response variables for analysis. We calculated the mean behaviour and hierarchy position (number of winning, dominant encounters) before and after treatment to obtain a robust estimate for brood care, aggression, ‘boldness’, activity and sociability (ants in close proximity and distance to the nearest ant) and position in the hierarchy before and after treatment. We then calculated the change in behaviour in response to treatment by subtracting the averaged behaviour value before treatment from the average value after treatment; positive values therefore indicate an increase and negative values a decrease in response to treatment. The same calculation was performed for the change in rank (number of encounters won in dyadic interactions). To further explore the qualitative differences between the treatments, we performed a one-way similarity of percentage (SIMPER) analysis, a data exploration technique that calculates the contributions individual factors make to both group (treatment) coherence and separation in a multidimensional scaling (MDS) analysis.

## Results

**Figure 1:**
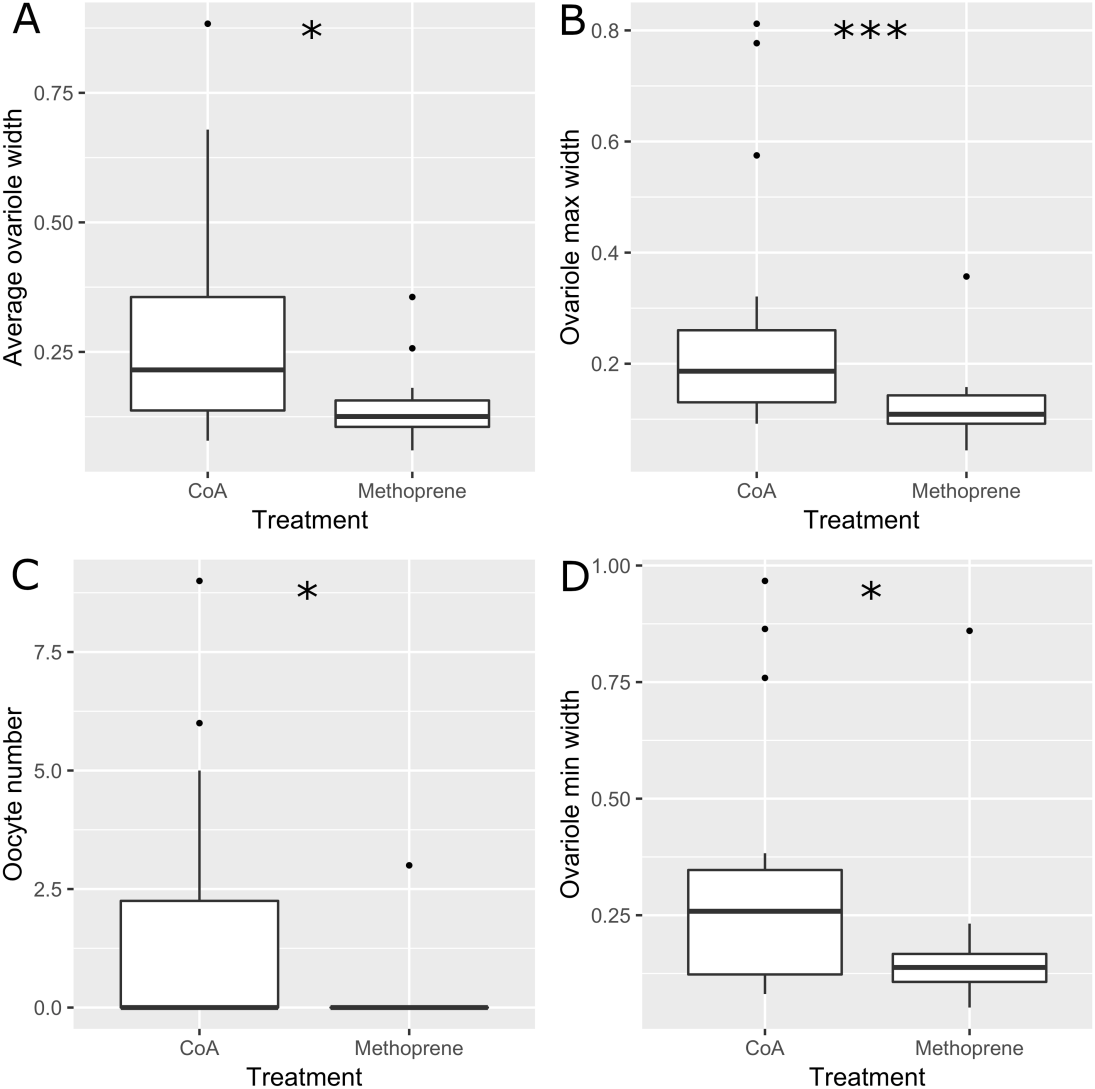
Boxplots showing fertility estimators measured in 32 *Dinoponera quadriceps* high ranked workers. Half of the ants were treated with Juvenile Hormone analogue (Methoprene) and half with acetone control (CoA). Fertility measures were the average ovariole width (A), maximum ovariole width (B), number of oocytes (C) and minimum ovariole width (D). Stars above plots indicate significant differences following Wilcoxon tests.

**Figure 2:**
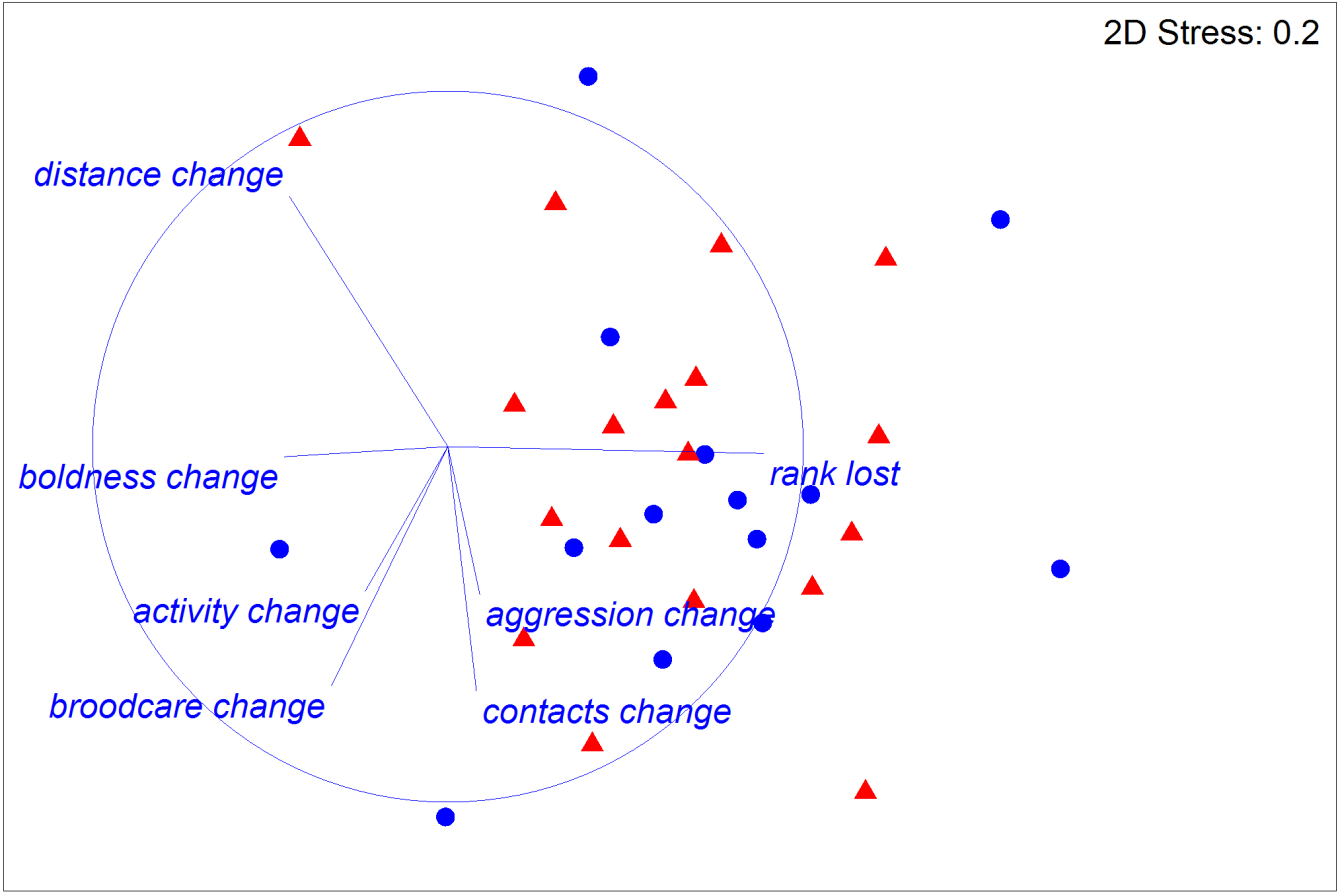
Multidimensional scaling (MDS) plot of all behaviours measured in 32 *Dinoponera quadriceps* high-ranked workers. Half of the ants were treated with Juvenile Hormone analogue (JHa; red triangles) and half with acetone control (CoA; blue circles). Behaviours measured were brood care, sociability as distance from nearest ant and number of ant contacts, activity, ‘boldness’ and aggression, as well as the change in rank following treatment. Vector lines indicate the strength and contribution of the individual traits for group separation between the two treatment groups. There were no significant differences between the treatments.

The experimental ants did not differ in size or weight between treatments or colonies (respectively: Pseudo *F*_1,31_ = 0.35, *P* = 0.72; Pseudo *F*_11,31_ = 1.04, *P* = 0.4; Table 1), and worker fertility also did not differ between colonies (Pseudo *F*_11,31_ = 1.82, *P* = 0.11). However, worker fertility was affected by treatment, with JHa-treated individuals being less fertile compared to those treated with acetone control under all measures of fertility taken here (oocyte number, average ovariole width, minimum ovariole width and maximum ovariole width; 22; Tables 1 and 2; Fig. 1). The CoA ants were more variable in fertility than the JHa group (Fig. 1). Furthermore, there were smaller differences in fertility within the JHa group (Fig. 1). In contrast to the effects of JHa treatment on worker fertility, we found no significant differences between treatment or colonies on social dominance behaviour (respectively: Pseudo *F*_1,31_ = 0.74, *P* = 0.6; Pseudo *F*_11,31_ = 1.37, *P* = 0.06; Fig. 2).

**Table 1:** Median values for physiological traits between acetone solvent control (CoA) individuals and methoprene treated Dinosaur ant individuals.

	**Weight [µg]**	**Head width [mm]**	**Ovariole max width**	**Ovariole min width**	**Oovariole average width**	**Oocyte number**
**CoA**	335	4.9	0.186	0.247	0.208	0
**Methoprene**	374	4.959	0.1075	0.142	0.122333334	0

**Table 2:** Results of the fertility Wilcoxon analyses comparing Dinosaur ant individuals treated with either acetone solvent control (CoA) or methoprene. Presented are the *W* test statistic and *P* values for each test carried out.

	***W***	***P*-value**
Oocyte number	187.5	0.015
Ovariole width (minimum)	200	0.021
Ovariole width (maximum)	212.5	0.006
Ovariole width (average)	202.5	0.017

## Discussion

Our results show that JH has a role in regulating reproduction in *D. quadriceps*. Experimentally elevated JH levels not only decreased the number of vitellogenic eggs in high-ranked workers, but also resulted in an overall decrease in the size of individual ovarioles, indicating a substantial reduction in reproductive potential. In solitary insects JH often has the opposite effect by stimulating the production of vitellogenic oocytes and the same is true for many primitively eusocial insects, such as non-swarm founding wasps and bumblebees (Robinson & Vargo, 1997). It has been suggested that the functional reversal of JH in reproduction was important in the evolution of complex societies, and a large number of studies demonstrate often radical changes in the regulatory architecture of reproduction in eusocial species (Robinson & Vargo, 1997; Hartfelder, 2000; Bloch et al., 2009). The classic example for this argument is the remodelling of the regulatory function of JH in honeybees and some ants (Robinson & Vargo, 1997; Bloch et al., 2009; Pamminger et al., 2016; Azevedo et al., 2016; Pamminger, Treanor & Hughes, 2016), however a small number of studies clearly indicates that high social organization is possible without it (e.g., Brent & Vargo, 2003; Kelstrup, Hartfelder & Wossler, 2015). *D. quadriceps* supports the former findings by demonstrating that a remodelling of JH function which inhibits reproduction can also be associated with simple social organization. This makes sense given the evolutionary position of *D. quadriceps*, with *Dinoponera* having secondarily reverted to simple, queenless societies from a highly eusocial ancestor (Peeters, 1997; Monnin & Peeters, 1998; Monnin & Peeters, 1999). This further supports the notion that there is likely no causal link between the remodelling of the JH function in reproduction and the organisational complexity of insect societies.

The results presented here, that JH significantly decreases fertility, are therefore consistent with those for other gamergate-led ant societies (*Harpegnathos*, *Streblognathus* and *Diacamma*; Sommer, Hölldobler & Rembold, 1993; Cuvillier-Hot, Lenoir & Peeters, 2004; Penick, Liebig & Brent, 2011). The role of JH in such societies is thus relatively clear, but there remain questions on how JH functions in true queens of other species. Importantly in these societies JH appears to be involved as part of an ‘honest’ signal that informs other colony members about their fertility (Cuvillier-Hot, Lenoir & Peeters, 2004), a key cue for the maintenance of dominance hierarchies in these societies. In studies on a gamergate ant society, a social wasp and a termite, topical applications of JH affect cuticular hydrocarbon (CHC) profiles of adult and larval individuals such that the profile becomes more ‘reproductive-like’ and is perceived so by colony members (Kelstrup et al., 2014; Brent et al., 2016; Penick & Liebig, 2017). However in *Streblognathus* ants alphas treated with JH had their fertility reduced and the CHC profile matched more closely with that of a sterile worker than an alpha (Cuvillier-Hot et al., 2004)

In contrast to the relatively well-studied effects of JH on reproductive physiology, little is known about the regulatory role of JH in behaviour for most insects. The association between JH and aggression, maternal behaviour and activity has been documented in insects (Nijhout, 1998; Pearce, Huang & Breed, 2001; Tibbetts & Izzo, 2009; Tibbetts, Vernier & Jinn, 2013), but these studies are restricted to only a handful of species. In the honey bee *Apis mellifera*, JH, in combination with the yolk precursor vitellogenin*,* regulates one of the major behavioural transitions in the adult honeybee worker from within-nest behaviour to external foraging (Robinson & Vargo, 1997). This transition is associated with a major remodelling of the behaviour repertoire and indicates the far-reaching regulatory potential of JH in behaviour. A similar function of JH has been documented in *Pogonomyrmex californicus* harvester ants and *Acromyrmex echinator* leaf-cutting ants (Dolezal et al., 2009; Norman & Hughes, 2016), demonstrating that JH can generate forager-like behavioural phenotypes. In contrast to our expectations, we find no measurable effects of JH on worker behaviour or position in the hierarchy in *D. quadriceps*. This could indicate that JH, fertility and dominance are decoupled in *D. quadriceps*. Indeed, a lack of behavioural effect of JHa on alphas in *Streblognathus* ponerine ants has been reported previously (Cuvillier-Hot et al., 2004). However, it is more likely that our study simply lacked the power to detect an effect. Our study was deliberately focussed on only high-ranked workers because these were the individuals in which both positive and negative effects could potentially be seen, and it may be that inclusion of ants from the full spectrum of the social hierarchy or using in-nest behavioural observations may reveal effects.

## Conclusions

Although JH is now known to have an important (though variable) role in the physiology of reproductive dominance in social insects, other hormones are also likely to be as, or more, important to reproductive status and social dominance in *D. quadriceps*. In particular, ecdysone or vitellogenin generate observed rank-specific phenotypes in other social insects (Hartfelder, 2000), and dopamine may also play a central role in the regulation of dominance and reproduction in species with simple societies (Sasaki, Yamasaki & Nagao, 2007; Okada et al., 2015; Ohkawara & Aonuma, 2016). Further work combining behavioural, genetic and physiological work is needed to illuminate the regulatory underpinning of reproductive hierarchies in simple ant societies. When looking at the broader phylogenetic picture there is accumulating evidence that JH occupies a stunning range of different, often opposite, regulatory functions. The question of how such incredible regulatory flexibility is possible without compromising fitness-relevant functions is intriguing and a promising target for further molecular and comparative investigations.

##  Supplemental Information

10.7717/peerj.6512/supp-1 Table S1 JHa doses used in previous social insect studies including bees, wasps and ants References 1. Tibbetts E. A., Vernier C., Jinn J. (2013). Juvenile hormone influences precontest assessment behaviour in *Polistes dominulus* paper wasps. Anim Behav*.* 85, 1177–1181. 2. Gamboa GJ, Noble MA, Thom MC, Togal JL, Srinivasan R, Murphy BD. 2004. The comparative biology of two sympatric paper wasps in Michigan, the native *Polistes fuscatus* and the invasive *Polistes dominulus* (Hymenoptera, Vespidae). Insectes Soc. 51: 153–157. 3. Norman VC, Hughes WOH. (2016). Behavioural effects of juvenile hormone and their influence on division of labour in leaf-cutting ant societies. * J Exp Biol*. 219: 8-11. 4. Cahan, SH, Graves CJ, Brent CS. 2011. Intergenerational effect of juvenile hormone on offspring in *Pogonomyrmex* harvester ants. J Comp Physiol B. 181: 991–999. 5. Pamminger T, Treanor D, Hughes WOH. 2016. Pleiotropic effects of juvenile hormone in ant queens and the escape from the reproduction–immunocompetence trade-off. Proc. R. Soc. B 283: 20152409 6. Robinson GE. 1985. Effects of a juvenile hormone analogue on honey bee foraging behaviour and alarm pheromone production. J Insec Physiol. 31: 277–282. 7. Cameron SA, Robinson GE. 1990. Juvenile hormone does not affect division of labor in bumble bee colonies (Hymenoptera: Apidae). Annal Entomol Soc Amer 83: 626–631.

10.7717/peerj.6512/supp-2 Supplemental Information 1 Raw data Raw data for Pamminger et al.

## Additional Information and Declarations

### Field Study Permissions

The following information was supplied relating to field study approvals (i.e., approving body and any reference numbers): Instituto Brasileiro do Meio Ambiente e dos Recursos Naturais (IBAMA) provided permit number 14BR004553 for this study.

### Data Availability

The following information was supplied regarding data availability: Raw data for Norman et al. are available as a Supplemental Figure.

## References

[ref-1] Anderson MJ, Gorley RN, Clarke KR (2008).

[ref-2] Asher CL, Nascimento FSF, Sumner S, Hughes WOH (2013). Division of labour and risk taking in the dinosaur ant, *Dinoponera quadriceps* (Hymenoptera: Formicidae). Myrmecological News.

[ref-3] Azevedo DO, De Paula SO, Zanuncio JC, Martinez LC, Serrão JE (2016). Juvenile hormone downregulates vitellogenin production in *Ectatomma tuberculatum* (Hymenoptera: Formicidae) sterile workers. Journal of Experimental Biology.

[ref-4] Bloch G, Shpigler H, Wheeler D, Robinson G (2009). Endocrine influences on the organization of insect societies. Hormones.

[ref-5] Bourke AF, Green HA, Bruford MW (1997). Parentage, reproductive skew and queen turnover in a multiple-queen ant analysed with microsatellites. Proceedings of the Royal Society B.

[ref-6] Brent C, Peeters C, Dietemann V, Crewe R, Vargo E (2006). Hormonal correlates of reproductive status in the queenless ponerine ant, *Streblognathus peetersi*. Journal of Comparative Physiology. A, Sensory, Neural, and Behavioral Physiology.

[ref-7] Brent CS, Penick CA, Trobaugh B, Moore D, Liebig J (2016). Induction of a reproductive-specific cuticular hydrocarbon profile by a juvenile hormone analog in the termite *Zootermopsis nevadensis*. Chemoecology.

[ref-8] Brent CS, Vargo EL (2003). Changes in juvenile hormone biosynthetic rate and whole body content in maturing virgin queens of *Solenopsis invicta*. Journal of Insect Physiology.

[ref-9] Cant MA, Llop JB, Field J (2006). Individual variation in social aggression and the probability of inheritance: theory and a field test. The American Naturalist.

[ref-10] Cuvillier-Hot V, Lenoir A, Crewe R, Malosse C, Peeters C (2004). Fertility signalling and reproductive skew in queenless ants. Animal Behavior.

[ref-11] Cuvillier-Hot V, Lenoir A, Peeters C (2004). Reproductive monopoly enforced by sterile police workers in a queenless ant. Behavioral Ecology.

[ref-12] Dolezal AG, Brent CS, Gadau J, Hölldobler B, Amdam GV (2009). Endocrine physiology of the division of labour in *Pogonomyrmex californicus* founding queens. Animal Behavior.

[ref-13] Field J, Solis CR, Queller DC, Strassmann JE (1998). Social and genetic structure of paper wasp cofoundress associations: tests of reproductive skew models. American Naturalist.

[ref-14] Gotelli NJ, Ellison AM (2004). A primer of ecological statistics.

[ref-15] Grainger DP, Asher CL, Jones JC, Nascimento FS, Sumner S, Hughes WOH (2014). A novel method of assessing dominance hierarchies shows nuance, linearity and stability in the dinosaur ant *Dinoponera quadriceps*. Ethology.

[ref-16] Hartfelder K (2000). Insect juvenile hormone: from “status quo” to high society. Brazilian Journal Of Medical and Biological Research.

[ref-17] Hölldobler B, Wilson EO (1990). The ants.

[ref-18] Jamieson IG (1997). Testing reproductive skew models in a communally breeding bird, the pukeko, *Porphyrio porphyrio*. Proceedings of the Royal Society B.

[ref-19] Johnstone R (2000). Models of reproductive skew: a review and synthesis (invited Article). Ethology.

[ref-20] Kelstrup HC, Hartfelder K, Nascimento FS, Riddiford LM (2014). The role of juvenile hormone in dominance behavior, reproduction and cuticular pheromone signaling in the caste-flexible epiponine wasp, *Synoeca surinama*. Frontiers in Zoology.

[ref-21] Kelstrup HC, Hartfelder K, Wossler TC (2015). *Polistes smithii* vs. *Polistes dominula*: the contrasting endocrinology and epicuticular signaling of sympatric paper wasps in the field. Behavioral Ecology and Sociobiology.

[ref-22] Kokko H (2003). Are reproductive skew models evolutionarily stable?. Proceedings of the Royal Society B.

[ref-23] Kokko H, Johnstone RA (1999). Social queuing in animal societies: a dynamic model of reproductive skew. Proceedings of the Royal Society B.

[ref-24] Libbrecht R, Corona M, Wende F, Azevedo DO, Serrao JE, Keller L (2013). Interplay between insulin signaling, juvenile hormone, and vitellogenin regulates maternal effects on polyphenism in ants. Proceedings of the National Academy of Sciences of the United States of America.

[ref-25] Monnin T, Peeters C (1998). Monogyny and regulation of worker mating in the queenless ant *Dinoponera quadriceps*. Animal Behavior.

[ref-26] Monnin T, Peeters C (1999). Dominance hierarchy and reproductive conflicts among subordinates in a monogynous queenless ant. Behavioral Ecology.

[ref-27] Monnin T, Ratnieks FLW, Brandão CRF (2003). Reproductive conflict in animal societies: hierarchy length increases with colony size in queenless ponerine ants. Behavioral Ecology and Sociobiology.

[ref-28] Monnin T, Ratnieks FLW, Jones GR, Beard R (2002). Pretender punishment induced by chemical signalling in a queenless ant. Nature.

[ref-29] Nascimento FS, Souza DISA, Tannure-Nascimento IC, Dantas JO (2012). Social facilitation and food partitioning in the queenless ant *Dinoponera quadriceps* (Hymenoptera: Formicidae). Journal of Natural History.

[ref-30] Neff B, Pitcher T, Ramnarine I (2008). Interpopulation variation in multiple paternity and reproductive skew in the guppy. Molecular Ecology.

[ref-31] Nijhout HF (1998). Insect hormones.

[ref-32] Norman VC, Hughes WOH (2016). Behavioural effects of juvenile hormone and their influence on division of labour in leaf-cutting ant societies. Journal of Experimental Biology.

[ref-33] Ohkawara K, Aonuma H (2016). Changes in the levels of biogenic amines associated with aggressive behavior of queen in the social parasite ant *Vollenhovia nipponica*. Insectes Sociaux.

[ref-34] Okada Y, Sasaki K, Miyazaki S, Shimoji H, Tsuji K, Miura T (2015). Social dominance and reproductive differentiation mediated by dopaminergic signaling in a queenless ant. Journal of Experimental Biology.

[ref-35] Pamminger T, Buttstedt A, Norman V, Schierhorn A, Botías C, Jones JC, Basley K, Hughes WOH (2016). The effects of juvenile hormone on *Lasius niger* reproduction. Journal of Insect Physiology.

[ref-36] Pamminger T, Foitzik S, Kaufmann KC, Schützler N, Menzel F (2014). Worker personality and its association with spatially structured division of labor. PLOS ONE.

[ref-37] Pamminger T, Treanor D, Hughes WOH (2016). Pleiotropic effects of juvenile hormone in ant queens and the escape from the reproduction-immunocompetence trade-off. Proceedings of the Royal Society B: Biological Sciences. B.

[ref-38] Patalano S, Vlasova A, Wyatt C, Ewels P, Camara F, Ferreira PG, Asher CL, Jurkowski TP, Segonds-Pichon A, Bachman M, González-Navarrete I, Minoche AE, Krueger F, Lowy E, Marcet-Houben M, Rodriguez-Ales JL, Nascimento FS, Balasubramanian S, Gabaldon T, Tarver JE, Andrews S, Himmelbauer H, Hughes WOH, Guigó R, Reik W, Sumner S (2015). Molecular signatures of plastic phenotypes in two eusocial insect species with simple societies. Proceedings of the National Academy of Sciences of the United States of America.

[ref-39] Pearce AN, Huang ZY, Breed MD (2001). Juvenile hormone and aggression in honey bees. Journal of Insect Physiology.

[ref-40] Peeters C, Choe JC, Crespi BJ (1997). Morphologically primitive ants: comparative review of social characters, and the importance of queen-worker dimorphism. The evolution of social behavior in insects and arachnids.

[ref-41] Penick CA, Liebig J (2017). A larval ‘princess pheromone’ identifies future ant queens based on their juvenile hormone content. Animal Behavior.

[ref-42] Penick CA, Liebig J, Brent CS (2011). Reproduction, dominance, and caste: endocrine profiles of queens and workers of the ant *Harpegnathos saltator*. Journal of Comparative Physiology. A, Sensory, Neural, and Behavioral Physiology.

[ref-43] Ratnieks FLW, Foster KR, Wenseleers T (2006). Conflict resolution in insect societies. Annual Review of Entomology.

[ref-44] Ratnieks FLW, Reeve HK (1992). Conflict in single-queen hymenopteran societies: the structure of conflict and processes that reduce conflict in advanced eusocial species. Journal of Theoretical Biology.

[ref-45] Reeve K, Keller L (2001). Tests of reproductive-skew models in social insects. Annual Review of Entomology.

[ref-46] Robinson GE, Vargo EL (1997). Juvenile hormone in adult eusocial hymenoptera: gonadotropin and behavioral pacemaker. Archives of Insect Biochemistry and Physiology.

[ref-47] Sasaki K, Yamasaki K, Nagao T (2007). Neuro-endocrine correlates of ovarian development and egg-laying behaviors in the primitively eusocial wasp (*Polistes chinensis*). Journal of Insect Physiology.

[ref-48] Smith CR, Suarez AV Tsutsui, ND, Wittman SE, Edmonds B, Freauff A, Tillberg CV (2011). Nutritional asymmetries are related to division of labor in a queenless ant. PLOS ONE.

[ref-49] Sommer K, Hölldobler B, Rembold H (1993). Behavioral and physiological aspects of reproductive control in a *Diacamma* species from Malaysia (Formicidae, Ponerinae). Ethology.

[ref-50] Tibbetts EA, Huang ZY (2010). The challenge hypothesis in an insect: juvenile hormone increases during reproductive conflict following queen loss in *Polistes* wasps. The American Naturalist.

[ref-51] Tibbetts EA, Izzo AS (2009). Endocrine mediated phenotypic plasticity: condition-dependent effects of juvenile hormone on dominance and fertility of wasp queens. Hormones and Behavior.

[ref-52] Tibbetts EA, Vernier C, Jinn J (2013). Juvenile hormone influences precontest assessment behaviour in *Polistes dominulus* paper wasps. Animal Behavior.

[ref-53] Tranter C, LeFevre L, Evison SEF, Hughes WOH (2014). Threat detection: contextual recognition and response to parasites by ants. Behavioral Ecology.

[ref-54] Widdig A, Bercovitch FB, Streich WJ, Sauermann U, Nürnberg P, Krawczak M (2004). A longitudinal analysis of reproductive skew in male rhesus macaques. Proceedings of the Royal Society B: Biological Science.

