# Supplementary material for: The role of juvenile hormone in regulating reproductive physiology and dominance in Dinoponera quadriceps ants
Source: PeerJ. 2019 Mar 1;7:e6512. doi: 10.7717/peerj.6512 (PMC6398374; doi:10.7717/peerj.6512)
Supplement: Table S1 — References 1. Tibbetts E. A., Vernier C., Jinn J. (2013). Juvenile hormone influences precontest assessment behaviour in Polistes dominulus paper wasps. Anim Behav. 85, 1177–1181. 2. Gamboa GJ, Noble MA, Thom MC, Togal JL, Srinivasan R, Murphy BD. 2004. The comparative biology of two sympatric paper wasps in Michigan, the native Polistes fuscatus and the invasive Polistes dominulus (Hymenoptera, Vespidae). Insectes Soc. 51: 153–157. 3. Norman VC, Hughes WOH. (2016). Behavioural effects of juvenile hormone and their influence on division of labour in leaf-cutting ant societies. J Exp Biol. 219: 8-11. 4. Cahan, SH, Graves CJ, Brent CS. 2011. Intergenerational effect of juvenile hormone on offspring in Pogonomyrmex harvester ants. J Comp Physiol B. 181: 991–999. 5. Pamminger T, Treanor D, Hughes WOH. 2016. Pleiotropic effects of juvenile hormone in ant queens and the escape from the reproduction–immunocompetence trade-off. Proc. R. Soc. B 283: 20152409 6. Robinson GE. 1985. Effects of a juvenile hormone analogue on honey bee foraging behaviour and alarm pheromone production. J Insec Physiol. 31: 277–282. 7. Cameron SA, Robinson GE. 1990. Juvenile hormone does not affect division of labor in bumble bee colonies (Hymenoptera: Apidae). Annal Entomol Soc Amer 83: 626–631. [file peerj-07-6512-s001.docx]

The ambiguous role of juvenile hormone in regulating reproductive physiology but not dominance hierarchy in *Dinoponera quadriceps*

| Table S1: JHa doses used in previous social insect studies including bees, wasps and ants. | | | | |  |
| --- | --- | --- | --- | --- | --- |
|  |  |  |  |  |  |
| Species | Weight [mg] | Jha [µg] | Jha [μg/mg] | Citation |  |
|  |  |  |  |  |  |
| *Polistes dominulus* | 90mg | 5μg | 0.060 | 1 |  |
| *Polistes dominulus* | 90mg | 25μg | 0.278 | 2 |  |
| *Acromyrmex octospinosus* | 13.43mg | 3.3μg | 0.223 | 3 |  |
| *Pogonomyrmex* J line | 16mg | 16μg | 0.625 | 4 |  |
| *Lasius niger* (queen) | 27mg | 1.1μg | 0.041 | 5 |  |
| *Apis mellifera* | 90mg | up to 250μg | 2.770 | 6 |  |
| *Bombus impatiens* | 200mg | 250μg | 1.250 | 7 |  |
| *Bombus bimaculatus* | 200mg | 250μg | 1.250 | 7 |  |
| *Dinoponera quadriceps* | 350mg | 16.5μg | 0.047 | This study |  |
|  |  |  |  |  |  |

References

1. Tibbetts E. A., Vernier C., Jinn J. (2013). Juvenile hormone influences precontest assessment behaviour in *Polistes dominulus* paper wasps. Anim Behav*.* **85**, 1177–1181.

2. Gamboa GJ, Noble MA, Thom MC, Togal JL, Srinivasan R, Murphy BD. 2004. The comparative biology of two sympatric paper wasps in Michigan, the native *Polistes fuscatus* and the invasive *Polistes dominulus* (Hymenoptera, Vespidae). Insectes Soc. 51: 153–157.

3. Norman VC, Hughes WOH. (2016). Behavioural effects of juvenile hormone and their influence on division of labour in leaf-cutting ant societies.  *J Exp Biol*. 219: 8-11.

4. Cahan, SH, Graves CJ, Brent CS. 2011. Intergenerational effect of juvenile hormone on offspring in *Pogonomyrmex* harvester ants. J Comp Physiol B. 181: 991–999.

5. Pamminger T, Treanor D, Hughes WOH. 2016. Pleiotropic effects of juvenile hormone in ant queens and the escape from the reproduction–immunocompetence trade-off. Proc. R. Soc. B 283: 20152409

6. Robinson GE. 1985. Effects of a juvenile hormone analogue on honey bee foraging behaviour and alarm pheromone production. J Insec Physiol. 31: 277–282.

7. Cameron SA, Robinson GE. 1990. Juvenile hormone does not affect division of labor in bumble bee colonies (Hymenoptera: Apidae). Annal Entomol Soc Amer 83: 626–631.
